# First observation of *Dorylus* ant feeding in Budongo chimpanzees supports absence of stick-tool culture

**DOI:** 10.1007/s10329-016-0533-3

**Published:** 2016-04-02

**Authors:** Steven Mugisha, Klaus Zuberbühler, Catherine Hobaiter

**Affiliations:** Budongo Conservation Field Station, PO Box 362, Masindi, Uganda; Department of Comparative Cognition, Institute of Biology, University of Neuchatel, 2000 Neuchâtel, Switzerland; School of Psychology and Neuroscience, University of St Andrews, St Mary’s College, South Street, St Andrews, Fife, KY16 9JP UK

**Keywords:** Tool use, Chimpanzee, *Pan troglodytes schweinfurthii*, Culture

## Abstract

The use of stick- or probe-tools is a chimpanzee universal, recorded in all long-term study populations across Africa, except one: Budongo, Uganda. Here, after 25 years of observation, stick-tool use remains absent under both natural circumstances and strong experimental scaffolding. Instead, the chimpanzees employ a rich repertoire of leaf-tools for a variety of dietary and hygiene tasks. One use of stick-tools in other communities is in feeding on the aggressive *Dorylus* ‘army ant’ species, consumed by chimpanzees at all long-term study sites outside of mid-Western Uganda. Here we report the first observation of army-ant feeding in Budongo, in which individuals from the Waibira chimpanzee community employed detached leaves to feed on a ground swarm. We describe the behaviour and discuss whether or not it can be considered tool use, together with its implication for the absence of stick-tool ‘culture’ in Budongo chimpanzees.

## Introduction

Chimpanzees are among a very limited group of species that not only employ, but also manufacture their own tools. Outside of our own, their tool use incorporates the richest range of tool types and techniques, including the use of composite tools (e.g., Sugiyama 1998), and tool kits with tools used in sequential order (e.g., Sanz and Morgan 2007). This rich repertoire has provided the strongest evidence for the occurrence of material culture in non-human animals; multiple behaviour variants within a community, transmitted socially between individuals (McGrew 1992; Whiten et al. 1999).

**Fig. 1:**
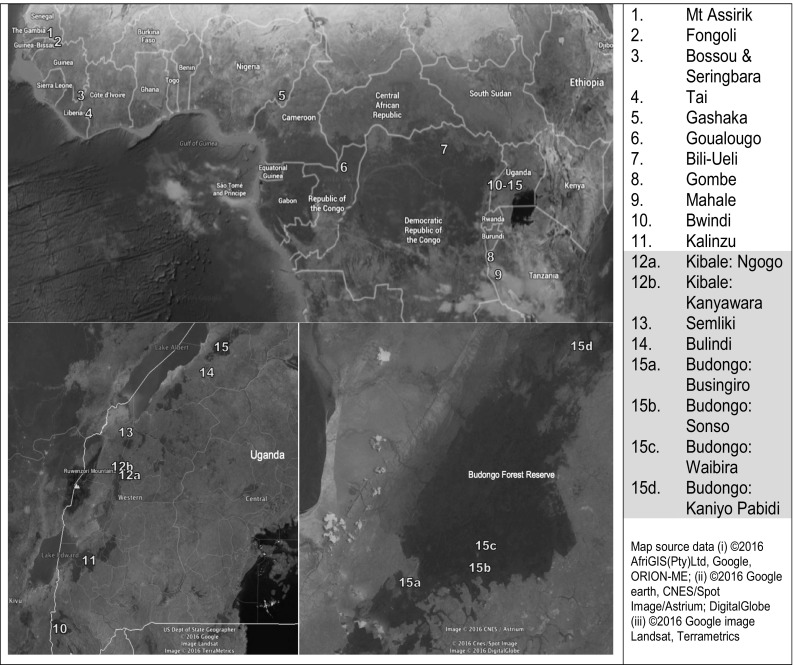
Location of chimpanzee study sites with published records of tool use and/or insectivory. *Sites 2-4, 6, 8, 9, 11, 12a* and *b, *and* 15b* are classed as long-term (study durations > 10 years). *Sites 12–15* are classed as the mid-Western Ugandan population. Section i (*top panel*) shows site locations across sub-Saharan Africa; section ii (*bottom left panel*) shows a detail map of study sites within Uganda; and section iii (*bottom right panel*) shows study sites in the Budongo Forest Reserve, Uganda

The now famous first descriptions of tool use by wild chimpanzees were the probe-tools employed at Gombe to feed on termites (van Lawick-Goodall 1968). Subsequently, stick-tool and probe-tool use has been recorded at all long-term chimpanzee study sites, with one exception: the Budongo forest (see Fig. 1). Here a 25-year study of the Sonso community has revealed a rich repertoire of leaf-tools employed for a range of tasks such as leaf-sponges for drinking and wound cleaning, leaf-napkins used to wipe genitals, and leaf inspection of insects while grooming (Whiten et al. 1999; Reynolds 2005; Quiatt 2006; Hobaiter et al. 2014). However, probe-tool use, or stick-tool use of any kind, has never been observed (Reynolds 2005; Gruber et al. 2011). This absence has persisted despite persistent attempts at experimental scaffolding of the behaviour (Gruber et al. 2011) and the habitual use of stick-tools in neighbouring forest fragments under 20 km from the main Budongo forest block (McLennan 2011).

Similarly, insectivory—a widespread behaviour through out most chimpanzee populations—appears to be rare in Budongo, where it is limited to occasional consumption of termites (within termite soil), and rare reports of feeding on caterpillars, wasp larvae, or weevils (Newton-Fisher 1999; Reynolds 2005). Ant-feeding has never been observed in the Sonso community, nor are ant remains found in faeces (collected as part of on-going chimpanzee health monitoring, as well as for DNA and dietary analyses, e.g., Reynolds 2005; Hedges and McGrew 2012). In fact, only a single ant-feeding observation exists for a Budongo chimpanzee: a Busingiro community adult male feeding opportunistically on arboreal ants (unknown sp.) climbing across his arm in 1962 (Reynolds 2005).

Insectivory is also rare in Kibale forest chimpanzees, and the Ngogo community exhibit the same apparent absence of ant-feeding as Sonso (Watts et al. 2012). However, insect consumption, including ant-feeding, is common in other mid-western areas of Uganda (see Fig. 1). In both the Semliki (Webster et al. 2014) and Bulindi (McLennan 2014) chimpanzees, the communities specialize on the less-aggressive arboreal weaver ants (*Oecophylla longinoda*). Only the south-western Ugandan chimpanzee population is known to feed on the aggressive army ants (*Dorylus sp.;* Kalinzu: Hashimoto et al. 2000; Bwindi: Stanford and Nkurunungi 2003). Here, they employ the typical long chimpanzee probe-tools used by all other long-term study populations outside of mid-western Uganda (Bossou and Seringbara: Mobius et al. 2008; Humle and Matsuzawa 2002; Fongoli: McGrew et al. 2005; Pruetz 2006; Gashaka: Fowler and Sommer 2007; Gombe: McGrew 1974; Goualougo: Sanz et al. 2004, 2009; Mahale: Nishida and Hiraiwa 1982; Mt Assirik: McGrew et al. 1979; Tai: Boesch and Boesch 1990; Bili: Hicks, unpublished data).

When compared with chimpanzee diets in nearby Kibale, the Budongo chimpanzees’ diet includes a high diversity of food items, including non-native species, that may buffer against food shortages and reduce the necessity to exploit alternative food resources that require tools for extraction (Reynolds 2005; Gruber et al. 2012). However, recent examinations of chimpanzee ecology in West African populations have suggested that opportunity (i.e., availability of food species, tool material), as opposed to necessity (i.e., limited access to other resources), more directly impacts chimpanzee feeding behaviour (Koops et al. 2013). Given the availability of suitable prey and tool materials (Hedges and McGrew 2012; Watts et al. 2012), the low exploitation of insect resources in Budongo and Kibale chimpanzees has been suggested to result from their relatively limited range of tool types that focus—exclusively in the case of the Budongo chimpanzees—on leaf-tools (Whiten et al. 1999; Reynolds 2005; Quiatt 2006). Interestingly, both ant-feeding (on the less aggressive arboreal species) and stick-tool use (to dig out ground bee nests) are regularly observed in intermediate communities (Semliki, Webster et al. 2014; and Bulindi, McLennan 2014; both located between Kibale and Budongo and likely once part of the same continuous forest block). Together, the evidence suggests that the absence of ant-dipping for aggressive army ants in mid-Western Ugandan chimpanzees, and the absence of any stick-tool or probe-tool use in Budongo chimpanzees, may best be explained by a localized lack of cultural knowledge (Gruber et al. 2009, 2011; McLennan 2014).

Here we report the first observation of army-ant-feeding in Budongo, in which individuals from the Waibira chimpanzee community employed detached leaves to feed on a ground swarm.

## Methods

### Study site and subjects

The Budongo forest is a semi-deciduous tropical rain forest located along the western Rift Valley, Uganda at a mean altitude of 1050 m. The reserve comprises 793 km^2^ of forest and grassland, with 482 km^2^ of continuous forest cover (Eggling 1947). The Budongo Conservation Field Station (BCFS) consists of two research communities of chimpanzees, Sonso (75 individuals, work started 1990, fully habituated) and Waibira (est. 100 individuals, work started 2011, partially habituated).

### Data collection

Waibira chimpanzees are followed on a daily basis from 6am to 6.30pm by BCFS field assistants and researchers who record party composition, ranging behaviour, and the frequency and duration of key social behaviours; in addition to maintaining a log book in which a daily summary is written, including any events of special interest, injuries and illnesses, all hunting behaviour, and interactions with other species.

## Observation

*6November 2014.* At 7:15 am, field assistant MS located and followed a party of 19 individuals feeding on fruits of *Celtis durandii*. At 9:30 am the party started to travel to the northwest, entering a dense area of swamp-forest approximately 200 m northwest of the main trail system. The party moved through the swamp-forest towards an area of mixed forest; at the border between the forest types, they encountered a ground swarm of *Dorylus* (unknown species) ants that covered an area approximately 10 m in diameter, with the nest at the centre. Two adult male chimpanzees (BEN 21 years, MAP 31 years) were clearly visible; other individuals could be heard nearby, but were not in clear sight. Both males were using leaves to feed on the ants. Individual leaves that were already covered with the swarming insects were plucked from low foliage or from leaf litter on the ground, and then quickly ‘swiped through’ the mouth. The leaf was then discarded and another selected. A second method of consumption employed was the use of a finger to bend a young sapling over towards them, picking the ants off directly with their mouths before jumping back away from the swarm. The party continued to feed on the ants for approximately 20 min before travelling north; MS lost sight of the party before rejoining them at 10:20 am as they fed on fruits of *Ficus sur*, where they remained feeding until 11:18 am.

## Discussion

Anecdotal evidence, particularly single records, should always be used with caution when interpreting animal behaviour. But, when properly recorded, such evidence can contribute to our understanding (Bates and Byrne 2007). We observed two Waibira chimpanzees employing at least two strategies to harvest army ants while avoiding being bitten. These included plucking leaves from low foliage or detached leaves from the ground and using the characteristic rapid swipe through the mouth action also employed when ant-dipping with probe-tools (McGrew 1974), as well as eating them directly from the foliage. Both techniques would be considered ‘direct-mouthing,’ as in neither case was the hand used to accumulate the ants before feeding. However, unlike the typical probe-tool technique, leaves were not then reinserted into the swarm to re-harvest more ants, but were instead discarded and another leaf already covered was selected. The re-insertion of a tool is regularly employed by Budongo chimpanzees when sponging for water (Hobaiter et al. 2014) or honey (Gruber et al. 2009), and while it is not known if this action pattern would transfer to ant-feeding, it is a part of their regular tool-use repertoire.

A factor that may have promoted the selection of a new leaf as opposed to re-insertion was that the ants were swarming over a large area (and numbers of leaves), rather than organised into a column. Ant-dipping typically occurs at nests or trails (Humle and Matsuzawa 2002; Mobius et al. 2008), allowing the chimpanzees to sit or stand away from the aggressive insects, whereas in this case, they had to regularly and rapidly shift position to avoid being bitten. Nevertheless, despite the apparent discomfort of the bites, the party remained feeding for 20 min. Under these conditions, it may be preferable to pick up ‘pre-loaded’ leaves from within the swarm, rather than re-insert a ‘used’ empty leaf and wait for new insects to climb onto it.

Can we consider the use of detached leaves in this context as tool-use? If so, it would fit well within the established Budongo repertoire in which the customary use of detached tools is limited to those made of leaves (Whiten et al. 1999). The definition of a tool is not straightforward and has varied substantially within the animal literature (c.f. Van Lawick-Goodall 1970; with Beck 1975). Schumaker et al. (2011) summarise the recent animal tool-use literature and define a tool as: “the external employment of an unattached or manipulable attached environmental object to alter more efficiently the form, position, or condition of another object, another organism, or the user itself, when the user holds, and directly manipulates the tool during or prior to use and is responsible for the proper and effective orientation of the tool” (Schumaker et al. 2011 p5). Following this definition, the behaviour would be classified as tool use. The detached leaves were unattached objects, used to alter the position of another organism (the ants), they were held by the user and were directly manipulated during use, during which the chimpanzees were responsible for their proper and effective orientation (for example: in the ‘swipe through’ action used to transfer the ants to the mouth). However, unlike in other forms of leaf-tool use, such as leaf-sponging, leaf-mopping, leaf-grooming, leaf-spooning, etc., the object/organism transferred/manipulated was already on the ‘tool’: the ants were swarming over a large area, so the leaves were ‘pre-loaded’ with the prey. Given this variation from typical leaf-tool use, we remain cautious about its classification as a tool.

Whether this behaviour is described as tool-use or a new form of feeding on insects, it is the choice of material: detached leaves, to address an ecological challenge (transferring noxious army ant prey to the mouth) for which other chimpanzee groups employ long stick- or probe-tools that is revealing. Although the Waibira chimpanzees were only recently habituated, and it is likely that substantial elements of their behaviour and diet remain unknown, the absence of probe-tool use in a context (army-ant feeding) in which all other communities are known to employ probe-tools, together with the apparent absence of stick-tool and probe-tool use in the long-term neighbouring Sonso study community, as well as other all other studied communities within the Budongo forest block (Gruber et al. 2012), suggests that probe-tool use is also absent in the Waibira chimpanzees.

It is particularly difficult to establish the absence of behaviour. In the case of chimpanzee tool-use, the appearance of absence may be the result of tool-use being extremely rare, or limited by seasonality, prey availability, or other ecological variables. Furthermore, the apparent absence of probe-tool use in Waibira today does not necessarily mean that it was absent in the past, or prevent its acquisition in the future. Females transfer regularly between the Sonso and Waibira communities (four known Sonso individuals in the past 5 years); however, despite regular migration of females between neighbouring communities of chimpanzees, local traditions can be maintained (Humle and Matsuzawa 2004; Humle 2010; Luncz and Boesch 2014; Luncz et al. 2015; Koops et al. 2015), perhaps through rapid conformity to the new community’s behaviour (Luncz and Boesch 2014). Thus, it remains possible that stick-tool use of some kind may be present in Waibira.

However, the unusual technique employed: detached leaves and feeding from a swarm, together with the absence of any non-trivial evidence of ant-remains in faeces and the absence of any feeding or tool-use traces at ant nests [Sonso: Hedges and McGrew 2012; Waibira, Sonso, and Kamira: Hobaiter unpublished data (265 nests/mounds; 11.5 km transects)] strongly supports the absence of stick-tool culture in the Waibira chimpanzees.

This absence remains an enigma. The mid-Western forests of Uganda were likely continuous only 8,000–10,000 years ago (Reynolds 2005). Today we see a complex pattern of presence and absence of both large behavioural categories—e.g., the total absence of stick and probe-tool use in Budongo—together with more subtle variation, e.g., the use of stick-tools for probing in Kibale, and for digging in Bulindi. Army-ant feeding is present in southwestern Uganda, but appears effectively absent through out the mid-Western population. This absence exists despite habitual feeding on arboreal ants, the readily available presence of both the ants and the materials for tool-making, and the apparent recognition of army ants as a desirable food resource. These patterns highlight the complexity of localized variation in chimpanzee behaviour that may emerge over a relatively short time-frame.

The use of stick-tools appears to be a chimpanzee species-typical behaviour, with cultural variation impacting the detail of the tool shape, length, or technique (McGrew 1992; Whiten et al. 1999; Luncz et al. 2015; Koops et al. 2015). Its complete absence in Budongo chimpanzees—even under natural circumstances that would promote it, such as army-ant feeding, or under strong experimental scaffolding, such as the honey-log—suggests the localized loss of this behaviour together with substantial resistance to its reacquisition or ‘re-innovation’ (Gruber 2013). While it is likely that, as is true today (e.g., Koops et al. 2015), minor variation existed between neighbouring communities prior to the fragmentation of the mid-Western forest block, it seems unlikely that this would explain the total absence of an otherwise chimpanzee-typical behaviour. A more parsimonious explanation would be the subsequent loss of some behaviour types in the Budongo population. We suggest that future research on the question of chimpanzee ‘cultural’ behaviour should consider not only classification of horizontal variation between sites, but also longitudinal emergence and disappearance of behavioural variation within populations.
